# Delayed risk stratification system in pT1aN0/Nx DTC patients treated without radioactive iodine

**DOI:** 10.1530/EC-17-0135

**Published:** 2017-08-18

**Authors:** Danuta Gąsior-Perczak, Iwona Pałyga, Monika Szymonek, Artur Kowalik, Agnieszka Walczyk, Janusz Kopczyński, Katarzyna Lizis-Kolus, Anna Słuszniak, Janusz Słuszniak, Tomasz Łopatyński, Ryszard Mężyk, Stanisław Góźdź, Aldona Kowalska

**Affiliations:** 1 Endocrinology ClinicHolycross Cancer Centre, Kielce, Poland; 2 Department of Molecular DiagnosticsHolycross Cancer Centre, Kielce, Poland; 3 Department of Surgical PathologyHolycross Cancer Centre, Kielce, Poland; 4 Laboratory of Tumor MarkersHolycross Cancer Centre, Kielce, Poland; 5 Department of Surgical OncologyHolycross Cancer Centre, Kielce, Poland; 6 Department of SurgeryOncology Center of Lublin Land, Lublin, Poland; 7 Cancer EpidemiologyHolycross Cancer Centre, Kielce, Poland; 8 Oncology ClinicHolycross Cancer Centre, Kielce, Poland; 9 The Faculty of Health SciencesJan Kochanowski University, Kielce, Poland

**Keywords:** delayed risk stratification system, differentiated thyroid cancer, early stage DTC, thyroid cancer

## Abstract

**Purpose:**

Delayed risk stratification (DRS) system by Momesso and coworkers was accepted by the American Thyroid Association as a diagnostic tool for the risk stratification of unfavorable clinical outcomes and to monitor the clinical outcomes of differentiated thyroid cancer (DTC) patients treated without radioactive iodine (RAI). The aim of this study was to evaluate the DRS system in patients with pT1aN0/Nx stage.

**Methods:**

The study included 304 low-risk patients after thyroidectomy (*n* = 202) or lobectomy (*n* = 102) without RAI and were treated at a single center. The median age was 50.5 years, 91.1% were women and the median follow-up was 4 years. DRS of the treatment response was performed based on medical records and according to the criteria of Momesso and coworkers. Disease course (recurrence, death) and status (remission, persistent disease) on December 31, 2016 were evaluated. The relationship between unfavorable outcomes and the DRS system was evaluated.

**Results:**

Response to initial therapy was excellent in 272 patients (89.5%), indeterminate in 31 (10.2%) and biochemical incomplete (increased TgAb levels) in one (0.3%). Two patients in the excellent response group experienced recurrence at 6 and 7 years of follow-up (after lobectomy). None of the patients with indeterminate and biochemical incomplete response developed structural disease, and none of the patients died during the follow-up.

**Conclusions:**

The DRS system was not useful for predicting the risk of unfavorable clinical outcomes and cannot be used to personalize the monitoring method of the disease in patients at pT1aN0/Nx stage who are not treated with RAI.

## Introduction

Increased access to imaging techniques and fine-needle aspiration cytology (FNAC) has resulted in an increase in the rate of detection of differentiated thyroid cancer (DTC) in recent years (1, 2, 3, 4). The increase in the number of new cases mostly affects low-stage papillary thyroid cancer (PTC), which responds well to treatment and has a low risk of recurrence (5, 6). These epidemiological changes modified the approach to treatment and monitoring of DTC. In many cases, lobectomy (L) is sufficient as surgical treatment and radioactive iodine (^131^I) therapy is often unnecessary (7). Much attention has been paid to personalizing the way the disease is monitored; however, research efforts have focused on patients at high risk of unfavorable clinical outcomes. Two systems for the early assessment of risk are currently used in clinical practice: a system adopted by the American Thyroid Association (ATA) and a slightly different system adopted by the European Thyroid Association (ETA) (8, 9). Neither system is perfect because of the high percentage of high-risk patients who respond well to treatment and are ultimately disease-free. In the 2016 ATA recommendations, delayed risk stratification (DRS) system was proposed by Momesso and coworkers (10). This system is based on the assessment of response to therapy 2 years after initial treatment and is considered the best method to predict the risk of unfavorable clinical outcome (7). The authors of the DRS system provided criteria for assessing treatment response in patients treated with total thyroidectomy (TT) and adjuvant ^131^I therapy as well as in patients treated exclusively with TT or L (11). Several centers validated the DRS method in patients treated with ^131^I (12, 13, 14, 15), whereas limited data are available on the validation of this system in patients who do not receive ^131^I treatment. The authors did not find a study evaluating the DRS system in patients with DTC at pT1aN0/Nx stage.

The aim of the present study was to evaluate the efficacy of the DRS method for predicting unfavorable clinical outcomes in patients with DTC at pT1aN0/Nx stage who were treated exclusively with L or TT without adjuvant ^131^I therapy.

## Materials and methods

### Patients and study design

Of 2100 DTC patients treated in a single center between 2000 and 2016, all patients who were not treated with ^131^I (*n* = 440) were initially included in the study. Patients with a follow-up period shorter than 24 months (a period necessary for DRS testing) were excluded from the study group (*n* = 68). Another group of 68 patients who had anti-thyroglobulin (Tg) antibodies (TgAb) monitored indirectly by a Tg recovery test, and not directly by TgAb immunoassay, were also excluded. Finally, 304 patients were included in the study.

The study plan was accepted by the Bioethics Committee at the Regional Chamber of Physicians.

### Treatment protocol and patient monitoring

Patients with a postoperative diagnosis of DTC underwent the first follow-up evaluation at 4–6 weeks after TT or L surgery. The procedures included a physical examination; neck ultrasound (US) and measurement of serum thyroid-stimulating hormone (TSH), Tg and TgAb levels. A whole body scan (WBS) was performed in patients who underwent TT. If the results indicated that the initial surgery was not radical enough, the patients were referred for secondary TT. Subsequent follow-up was performed every 6–12 months depending on the degree of risk of unfavorable clinical outcomes. The procedures included US of the thyroid bed, US of the cervical lymph nodes and measurement of serum TSH, Tg and TgAb levels. In patients released from the oncology center (between visits), the levothyroxine (LT_4_) dose was adjusted by the general practitioner to ensure that TSH levels were within the recommended range for the patient.

### Investigations

From 2000 until 2007, Tg concentrations were evaluated using a chemiluminescent assay on the Immulite 1 analyzer (Diagnostic Products Corporation, Los Angeles, CA, USA), which has an analytical sensitivity of 0.2 ng/mL. However, from May 2004 until June 2005 the analytical sensitivity was 0.5 ng/mL. After 2007, Tg concentrations were evaluated with the same method of chemiluminescence on the Immulite 2000 xpi Immunoassay System analyzer (Diagnostic Products Corporation/Siemens). The method has an analytical sensitivity of 0.2 ng/mL and a functional sensitivity of 0.9 ng/mL (15, 16). From 2000 until 2007, the concentration of anti-Tg (TgAb) was evaluated using a radioisotope assay (RIA) (BRAHMS), which had an analytical sensitivity of 5.5 U/mL and a functional sensitivity <20 U/mL. From January 2007 until May 2008, the TgAb concentration was measured using a Chemiluminescent Microparticle Immuno Assay CMIA on the Architect 2000 analyzer (Abbott) using a chemiluminescence method. After May 2008, TgAb concentration was measured using a chemiluminescence method on the Immulite 2000 xpi Immunoassay System analyzer (Diagnostic Products Corporation/Siemens). Analytical sensitivity: 2.2 IU/mL (15, 16). From 2000 until 2007, TSH concentrations were evaluated using a chemiluminescent assay on the Immulite 1 analyzer (Diagnostic Products Corporation), which has an analytical sensitivity of 0.002 µIU/mL. After 2007, TSH concentrations were evaluated with the same method of chemiluminescence on the Immulite 2000 xpi Immunoassay System analyzer (Diagnostic Products Corporation/Siemens). The method has an analytical sensitivity of 0.002 µIU/mL. From 2010, TSH concentrations were measured using a Chemiluminescent Microparticle Immuno Assay CMIA on the Architect 2000 analyzer (Abbott) using a chemiluminescence method with an analytical sensitivity of ≤0.0025 µIU/mL and a functional sensitivity of ≤0.01 µIU/mL. Neck US was performed using a Siemens Versa pro and a Hitachi EUB-6500 (both featuring a color Doppler function) with a high-frequency linear probe (7.5 MHz). WBS was performed with a Symbia T2 gamma camera (Siemens) using a high-energy collimator with a scanning speed of 10 cm/min. Diagnostic WBS was performed at 72 h after the administration of 180 MBq ^131^I (rhTSH) or 80 MBq ^131^I (TWD) (15, 16).

### Risk stratification systems

All DTC cases were staged postoperatively according to the 7th American Joint Committee on Cancer/Union for International Cancer Control (AJCC/UICC) tumor-node-metastasis (TNM) staging system and in accordance with the ATA (8, 17) initial risk stratification system. After 2015, response to treatment was evaluated and patients were reclassified 2 years after surgery according to the DRS system. The DRS risk level correlated with the frequency of follow-up examinations and the degree of TSH suppression in response to LT_4_ therapy. Patients included in the study who had not previously been evaluated according to the DRS were evaluated based on medical records. The treatment response criteria proposed by Momesso and coworkers (10) were as follows: excellent response (unstimulated Tg levels <0.2 ng/dL for TT and <30 ng/dL for L, stable in two consecutive assays with similar TSH levels, undetectable TgAb, and no evidence of disease (NED) on neck US); indeterminate response (unstimulated Tg levels 0.2–5.0 ng/dL for TT, TgAb detectable but stable or declining, and non-specific findings on neck US); biochemical incomplete response (unstimulated Tg levels >5.0 ng/dL for TT and >30 ng/dL for L, Tg increasing by at least 20% with similar TSH, TgAb increasing by at least 20%, and NED on neck US) and structural incomplete response (structural findings on neck US confirmed by FNAC or scintigraphy).

### End of follow-up with oncological assessment on 31.12.2016

Based on the medical records, the health status of patients was classified as NED, persistent disease, recurrence, death from cancer and death for other reasons. Biochemical data (Tg and TgAb) were not used as criteria for persistent disease status. Any additional treatment for DTC was also analyzed.

### Statistical analysis

The following statistical analysis models were used: continuous variables (mean, median, standard deviation and interquartile range) and categorical variables (presented as numbers with percentages) were determined. The Student’s *t*-test for normally distributed variables and the Mann–Whitney *U* test for non-normally distributed variables were used to compare continuous variables (lobectomy vs. thyroidectomy). A bivariate analysis (chi-square test) was used to compare categorical variables (type of treatment vs. response to treatment). Calculations were performed with MedCalc Statistical Software, version 17.2 (MedCalc Software bvba, Ostend, Belgium; https://www.medcalc.org, 2017).

## Results

### Characteristics of the study group

The clinicopathological characteristics of patients are summarized in Table 1. Tumors were slightly larger in patients who underwent TT (5.3 mm) than in the L group (4.6 mm), and lymph nodes were more frequently detected in the TT group than in the L group (63.4% vs 25.5%) on histopathological examination.

**Table 1:** Characteristics of DTC patients treated with total thyroidectomy or lobectomy without adjuvant radioactive iodine therapy.

**Characteristics**	**Total** (*n* = 304)	**Lobectomy** (*n* = 102)	**Total thyroidectomy** (*n* = 202)	***P*-Value**
Age (years) mean (s.d.)	49.9 (13.6)	49.8 (13.6)	50.3 (13.6)	0.6723
Sex. *n* (%)				
Male	27 (8.9%)	8 (7.8%)	19 (9.4%)	
Female	277 (91.1%)	94 (92.2%)	183 (90.6%)	0.6545
Histology				
PTC-CV	229 (75.3%)	74 (72.6%)	156 (77.2%)	
PTC-FV	72 (23.7%)	28 (27.5%)	43 (21.3 %)	
FTC	3 (1%)	0	3 (1.5%)	0.6457
Size of tumor (mm) mean (s.d.)	5.1 (2.5)	4.6 (2.3)	5.3 (2.6)	0.0444
Lymph node classification (%)				
N0	154 (50.7%)	26 (25.5%)	128 (63.4%)	
Nx	150 (49.3%)	76 (74.5%)	74 (36.6%)	<0.0001
Follow-up (months) mean (s.d.)	53 (38.4)	60 (40.8)	44 (33.6)	<0.0001

DTC, differentiated thyroid cancer; FTC, follicular thyroid cancer; PTC-CV, papillary thyroid cancer classic variant; PTC-FV, papillary thyroid cancer follicular variant; s.d., standard deviation.

### Evaluation of treatment response according to the DRS system

In the TT and L groups, 178 (88.1%) and 94 (92.1%) patients had an excellent response to treatment, and 24 (11.9%) and 7 (6.9%) patients had an indeterminate response, respectively. Only one patient in the L group met the criteria for biochemical incomplete response (increasing TgAb levels). There were no cases of structural incomplete response. Differences in the response to treatment were not dependent on the scope of the surgery (*P* = 0.1512, Table 2).

**Table 2:** Dynamic risk stratification based on response to the initial therapy in the study group.

**Response to the initial therapy**	**Lobectomy** (*n* = 102)	**Total thyroidectomy** (*n* = 202)	***P*-Value**
Excellent (*n* = 272)	94 (92.1%)	178 (88.1%)	
Indeterminate (*n* = 31)	7 (6.9%)	24 (11.9%)	
Biochemical incomplete (*n* = 1)	1 (1.0%)	0	
Structural incomplete (*n* = 0)	0	0	0.1512

### Analysis of additional interventions for PTC and final clinical outcome

During the median follow-up of 53 months (range, 24–120 months), there were two cases of recurrence, one nodal recurrence (lateral nodules on the side of the operated tumor) and one case of new PTC onset (in the remaining left lobe). Both patients underwent L, and after 2 years, they met the criteria for excellent response. PTC recurrences occurred after 6 and 7 years in the two patients and were diagnosed by US, confirmed by FNAC and successfully cured with repeat surgery. Tg concentration was stable in both cases at the time of recurrence (Table 3). During the follow-up period, none of the patients from the indeterminate response group had structural disease (Table 4). New focal lesions in the thyroid remnants, which were later confirmed to be benign by FNAC, were reported in two patients. One patient underwent surgical treatment at her request without a diagnosis of PTC on postoperative examination.

**Table 3:** TSH and Tg levels in patients with recurrence after a period of no evidence of disease (NED).

**Patient**	**SM**		**ZM**	
	TSH (µIU/mL)	Tg (ng/mL)	TSH (µIU/mL)	Tg (ng/mL)
After surgery	15.8	25.2	5.24	23.8
Follow-up	1.37	3.79	2.19	22.5
	0.07	2.88	1.25	7.28
	0.25	2.90	0.09	11.7
	0.10	4.45	1.34	8.32
	0.33	3.81	0.20	19.8
	0.26	3.29	0.58	12.6
At the time of recurrence	0.26	2.88	0.43	9.62

SM, patient 1; Tg, thyroglobulin; TSH, thyroid-stimulating hormone; ZM, patient 2.

**Table 4:** Clinical outcome at the end of follow-up according to the ATA and DRS systems.

**Stratification type**	**NED** ***n*** (%)	**Persistent structural disease**	**Recurrent disease**	**Tumor-related deaths**
ATA-LR	304 (100%)	0	2 (0.6%)	0
DRS excellent				
Lobectomy: 94	94 (100%)	0	2 (2.1%)	0
TT: 178	178 (100%)	0	0	0
DRS indeterminate				
Lobectomy: 7	7 (100%)	0	0	0
TT: 24	24 (100%)	0	0	0
DRS biochemical incomplete				
Lobectomy: 1	1 (100%)	0	0	0
TT: 0		0	0	0

ATA, American Thyroid Association; ATA-LR, low-risk patients according to the American Thyroid Association; DRS, dynamic restratification system; NED, no evidence of disease; TT, total thyroidectomy.

All patients were in remission at the end of the follow-up period. There were no deaths from cancer or other causes.

## Discussion

DTC epidemiology has changed in recent decades. Despite a significant increase in morbidity, mortality remains constant (18). An increasing number of patients who are treated for DTC are patients with PTC at a low clinical stage (5, 6). Many patients undergo L or TT surgery without adjuvant ^131^I (7). This treatment strategy is associated with reduced sensitivity and specificity of the primary DTC markers such as Tg and ^131^I scintigraphy. In 2014, Momesso & Tuttle proposed a DRS system for stratifying the risk of unfavorable clinical outcomes based on the response to initial treatment in patients treated with and without ^131^I (11). Several centers validated the system in patients after ^131^I treatment (12, 13, 14, 15) and demonstrated its superiority over the ATA or ETA initial risk stratification system, in which the response to treatment is evaluated shortly after surgery (8, 9). Two studies by Momesso and coworkers and Park and coworkers validated the DRS in patients who were not treated with ^131^I after TT and L (10, 19). The authors confirmed the usefulness of the DRS in the study groups and showed that the biochemical and structural incomplete response groups (10, 19) had a statistically significantly higher risk of persistent/recurrent disease than the excellent response group. The population evaluated by Park and coworkers was significantly different from the present cohort in terms of the initial stage of the disease, with 41% vs 0% patients with extrathyroidal extension, 26.6% vs 0% patients with N1a and 26.9% vs 0% patients with stage III in Park and coworkers (19) and in the present study, respectively. In terms of the treatment response, there were 8.4% vs 1% biochemical incomplete response patients and 1.4% vs 0% structural incomplete response patients in Park and coworkers (19) and in the present study, respectively. However, an indeterminate response was not associated with a greater risk of unfavorable clinical outcomes in both studies. In addition, both studies found a similar risk of disease recurrence in the group with excellent initial response, 0.8% in Park and coworkers (19) and 0.7% in the present study. All four recurrences had unchanged Tg levels, and one case in the study by Park and coworkers showed a sudden increase in serum TgAb levels (19). All recurrences were diagnosed by US and FNAC, and they occurred at 4–7 years after the initial treatment. These findings confirm the need for long-term follow-up of patients with DTC despite an excellent response to treatment and indicate the importance of US as a diagnostic measure. In the study by Momesso and coworkers, DTC recurrence was not observed in patients with excellent response to treatment; however, this may be attributed to the short period of observation and the relatively small group of patients (10). The DRS system is useful to identify patients with persistent disease who can be screened with an additional diagnostic method to locate the structural disease, whereas it is not useful to assess the risk of a true recurrence after the period of NED. None of the patients in the present study had persistent disease, which may be due to the characteristics of the group (initially very low stage of disease after surgical treatment). One patient who had increased TgAb at 2 years after surgery showed a decrease in TgAb during the follow-up, and she eventually qualified as NED at the end of the study. These factors affected the final assessment of the DRS, which was found to be of little value to predict the outcomes of DTC patients at pT1aN0/Nx stage. The identification of risk factors for recurrence of DTC therefore remains unresolved.

In conclusion, patients with pT1aN0/Nx-stage disease and a low risk of unfavorable clinical outcomes showed a good prognosis irrespective of the extent of TT or L surgery. The risk of disease recurrence in the present study was low (0.7%), although recurrence was possible regardless of the response to initial therapy (both recurrences in the present study occurred in patients with excellent response). The DRS system was not useful for predicting the risk of recurrence in patients with a low risk of unfavorable clinical outcome.

## Declaration of interest

The authors declare that there is no conflict of interest that could be perceived as prejudicing the impartiality of the research reported.

## Funding

This work did not receive any specific grant from any funding agency in the public, commercial, or not-for-profit sector.
